# Development of a Corpus Annotated With Mentions of Pain in Mental Health Records: Natural Language Processing Approach

**DOI:** 10.2196/45849

**Published:** 2023-06-26

**Authors:** Jaya Chaturvedi, Natalia Chance, Luwaiza Mirza, Veshalee Vernugopan, Sumithra Velupillai, Robert Stewart, Angus Roberts

**Affiliations:** 1 Department of Biostatistics and Health Informatics King's College London London United Kingdom; 2 Department of Psychological Medicine King's College London London United Kingdom; 3 College of Medical, Veterinary and Life Sciences University of Glasgow Glasgow United Kingdom; 4 South London and Maudsley Biomedical Research Centre London United Kingdom

**Keywords:** pain, mental health, natural language processing, annotation, information extraction

## Abstract

**Background:**

Pain is a widespread issue, with 20% of adults (1 in 5) experiencing it globally. A strong association has been demonstrated between pain and mental health conditions, and this association is known to exacerbate disability and impairment. Pain is also known to be strongly related to emotions, which can lead to damaging consequences. As pain is a common reason for people to access health care facilities, electronic health records (EHRs) are a potential source of information on this pain. Mental health EHRs could be particularly beneficial since they can show the overlap of pain with mental health. Most mental health EHRs contain the majority of their information within the free-text sections of the records. However, it is challenging to extract information from free text. Natural language processing (NLP) methods are therefore required to extract this information from the text.

**Objective:**

This research describes the development of a corpus of manually labeled mentions of pain and pain-related entities from the documents of a mental health EHR database, for use in the development and evaluation of future NLP methods.

**Methods:**

The EHR database used, Clinical Record Interactive Search, consists of anonymized patient records from The South London and Maudsley National Health Service Foundation Trust in the United Kingdom. The corpus was developed through a process of manual annotation where pain mentions were marked as relevant (ie, referring to physical pain afflicting the patient), negated (ie, indicating absence of pain), or not relevant (ie, referring to pain affecting someone other than the patient, or metaphorical and hypothetical mentions). Relevant mentions were also annotated with additional attributes such as anatomical location affected by pain, pain character, and pain management measures, if mentioned.

**Results:**

A total of 5644 annotations were collected from 1985 documents (723 patients). Over 70% (n=4028) of the mentions found within the documents were annotated as relevant, and about half of these mentions also included the anatomical location affected by the pain. The most common pain character was chronic pain, and the most commonly mentioned anatomical location was the chest. Most annotations (n=1857, 33%) were from patients who had a primary diagnosis of mood disorders (International Classification of Diseases—10th edition, chapter F30-39).

**Conclusions:**

This research has helped better understand how pain is mentioned within the context of mental health EHRs and provided insight into the kind of information that is typically mentioned around pain in such a data source. In future work, the extracted information will be used to develop and evaluate a machine learning–based NLP application to automatically extract relevant pain information from EHR databases.

## Introduction

Pain is a growing focus of research, especially since the opioid crisis in the United States [1]. Pain can have long-term implications on the emotional well-being and mental health of people [2] due to its debilitating nature and has a potential impact on health care and societal costs [3]. Pain is known to affect 1 in 5 people [4], is a common reason for people to access health care facilities, and therefore features in patients’ health records. For these reasons, a potential source of information on pain is electronic health records (EHRs), which contain rich data on interactions between clinicians and patients [5].

Mental health EHRs are particularly beneficial for this research since they have the potential to show the recorded overlap of pain with mental health in clinical encounters. EHRs generally consist of structured information (such as tables and forms) and unstructured information (such as correspondence letters and discharge summaries). In mental health EHRs, the majority of information lies within the unstructured and free-text sections of the records [5]. Free-text fields allow clinicians the flexibility required to capture pertinent information on patient experiences, which might not be possible in the structured fields, which contain mostly drop-down menus and predetermined options that would not fit every patient situation. A systematic review conducted by Le Glaz et al [6] found that EHR data were the most commonly used corpus type and highlighted the importance of using machine learning and natural language processing (NLP) methods to obtain information on unexplored patient mental health data. A similar review by Zhang et al [7] found an increasing trend in the use of NLP in the context of mental health research over the past decade.

Tian et al [8] have presented work in which they attempted to identify patients with chronic pain from EHRs in a primary care setting. They used structured information from the EHR, including a combination of diagnostic codes for potential chronic painful conditions, patient-reported pain scores, and opioid prescription medications to identify patients [8]. Their research has highlighted that pain is not captured very well in the coded structured fields of EHRs, thereby making the free-text sections a valuable resource to extract this information. However, since the description of pain is quite ambiguous in nature, it is challenging to extract accurate information about pain from text. Carlson et al [9] sought to identify chronic pain episodes from health records at the Mayo Clinic and the Olmstead Medical Centre. They used diagnosis codes from structured tables to identify a cohort of patients with chronic pain, in conjunction with free-text annotations to include dates, locations, severity, cause, and pain management measures [9]. However, structured fields might not always include relevant diagnosis codes, especially when pain is a symptom rather than a diagnosis. In their systematic review, Koleck et al [10] found that the primary emphasis in the field of NLP is currently on creating techniques to extract symptom information from EHR narratives. The review also highlights the significance of this research direction, given the growing burden on patients and health care systems related to symptoms. Naseri et al [11] developed a pipeline for extraction of physician-reported pain from clinical reports, where they assigned pain scores to the clinical notes based on some rule-based algorithms and then used these pain scores to label the notes as pain, no pain, or no mention of pain. A quantitative review by Tighe et al [12] explored a topic modeling and deep learning–based text generation approach, using pain-related PubMed abstracts to identify trend gaps in pain in research. NLP methods offer a potential solution, using computational methods for analysis of linguistic data, aiding in the identification and the efficient extraction of relevant pain information from clinical documents.

The aim of the research described here was to develop a corpus of mentions of pain from the documents of a mental health EHR database called CRIS (Clinical Record Interactive Search), which consists of anonymized patient records from The South London and Maudsley (SLaM) National Health Service Foundation Trust in the United Kingdom, one of the largest mental health care providers in Western Europe [13]. Documents containing mentions of pain were identified and manually annotated with a number of different pain attributes, thereby creating a human-labeled data set. In future work, these labeled documents will be used to develop an NLP application to automatically extract such information from EHR databases. Along with development of the annotated corpus, this paper also investigates the distribution of pain and its different attributes within these mental health records. To the best of our knowledge, such an extraction of pain information from clinical text of mental health EHRs has not previously been conducted.

## Methods

### Ethics Approval

The CRIS application [13] was approved as a database for secondary analysis by the Oxford Research Ethics Committee (18/SC/0372). The work undertaken as part of this project as well as related research, including COVID-19–related work, were all approved by the CRIS Oversight Committee (CRIS project: 21-021). Service users are actively involved in the development of the CRIS database and manage the strict governance frameworks related to it. The data extracted were deidentified, and all SLaM patients are given the opportunity to opt out of their data being used for purposes other than their care [14-16].

### Data Source

The CRIS data platform consists of deidentified records from SLaM, one of the largest mental health care providers in Western Europe [13]. It consists of trust-wide records from 2006 to date and is supported by the NIHR Biomedical Research Centre at SLaM and King’s College London [13]. CRIS follows a robust, patient-led governance model and has ethical approval for secondary analysis (Oxford C Research Ethics Committee, reference 18/SC/0372). The free text within CRIS is composed of progress notes, discharge summaries, written assessments, correspondence documents, and more. There are over 30 million case notes within this database, averaging about 90 documents per patient [17].

### Data Extraction

EHR structured tables and codes do not necessarily include information about pain, potentially due to it being a symptom rather than a diagnosis, making it difficult to extract documents based on codes alone. Therefore, this information was sought from the unstructured free-text fields of the database. A search was conducted on all the text sources within CRIS for the word “pain” to gauge where information about pain might be recorded most frequently. The majority of mentions of pain were in documents from CRIS “Event” and “Attachments” tables (Table 1), and so these were used in the next steps. In SLaM EHRs, “Event” documents represent conventional case notes, usually completed by the reviewing member of staff contemporaneously with, or shortly after, clinical contacts. “Attachment” documents contain formal clinical correspondence, most typically between the reviewing member of staff and the referring clinician (eg, the patient’s primary care physician).

To identify documents within these tables that might contain mentions of pain, a lexicon of pain terms was used. This lexicon was developed by combining multiple data sources, as described in full in reference [18]. The lexicon consists of 382 unique pain-related terms. Since running a query on a database with such a large number of terms would be computationally expensive, the list of terms was generalized using wild cards (%), such as %pain% to capture concepts like back pain, pains, %ache for headache, and so on. After creation of wild cards, 35 unique extraction terms were used in the query. Some of these terms are shown in Table 2. The intention was that these limited keywords would capture all the terms within the lexicon, albeit at the risk of a lower precision than the lexicon itself. This approach led to some false positives such as *paint*, *painting*, and *spain* for the wild card word %pain% and attached or attaches for %ache%. Ache was modified to multiple wild card terms such as %ache, %aches, achin%, in order to avoid picking up some of the common false-positive terms. If picked up, such false positives were eliminated during the manual annotation stage. Words that could not be converted into wild cards were used in their full form, such as “mittelschmerz,” “lumbago,” “migraine.”

**Table 1:** Common sources of text within the whole of CRIS^a^ and the count of documents with matched pain terms within each of these sources.

Text source	Pain terms matched within the documents, n
Event	1,063,523
Attachments	297,538
CAMHS^b^ event	36,857
Discharge notification summary	13,175

^a^CRIS: Clinical Record Interactive Search.

^b^CAMHS: Child and Adolescent Mental Health Services.

**Table 2:** Pain words with corresponding wild cards and examples.

Pain word	Word with wild card (%)	Example words
Pain	%pain%	Pains, painful
Ache	%ache	Headache, backache
Ache	%aches	Headaches, aches
Sore	sore%	Soreness, sores
Algesia	%algesi%	Analgesia, analgesic
Algia	%algia%	Proctalgia, neuralgias
Burn	%burn%	Heartburn, burns, burning
Colic	colic%	Colicky pain
Cramp	cramp%	Cramps, cramping
Dynia	%dynia%	Allodynia, glossodynia
Hurt	hurt%	Hurts, hurting
Rheumatic	rheumati%	Rheumatic, rheumatism
Sciatic	sciati%	Sciatic, sciatica
Spasm	spasm%	Spasms, spasmic
Tender	tender%	Tenderness

A SQL query was run to extract any documents that contained these keywords. No diagnosis or time filters were applied to the data extraction.

### Annotation Process

A small sample of 50 documents was extracted to examine the different contexts in which pain is mentioned. This was used to initiate the development of annotation guidelines. These guidelines were drafted to ensure consistent annotation by multiple annotators. Upon extraction, these documents were preannotated with pain terms (labeled as a mention of pain, as seen in Table 3) from the lexicon and loaded into an annotation tool, MedCAT [19], for manual verification and annotation of these mentions of pain. Three medical students were employed to manually verify these annotations as well as add any associated attributes (features associated with the labeled text) based on the context around the mention of pain.

**Figure 1:**
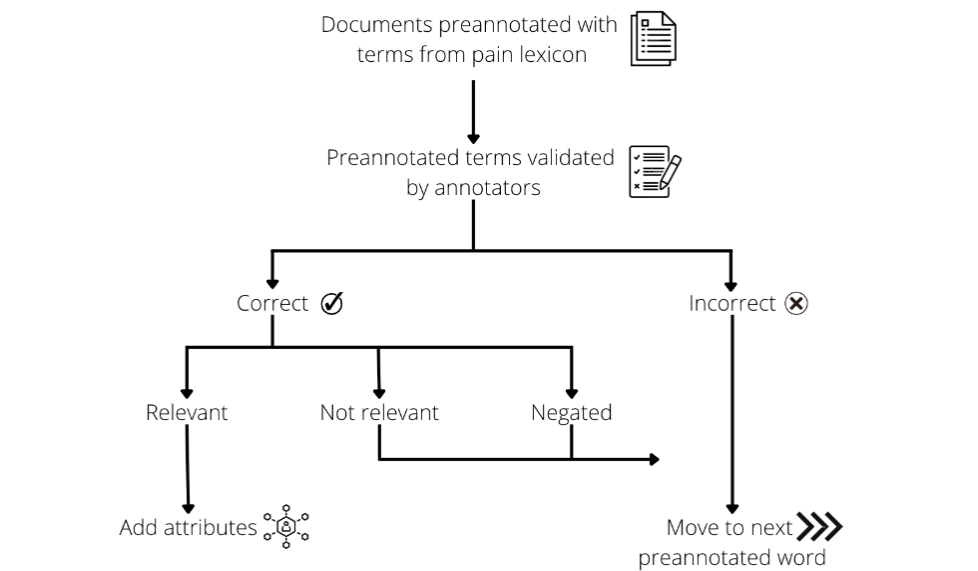
Annotation process.

The first rounds of annotation, which were for the purposes of refining the annotation guidelines and training the annotators, consisted of 4 rounds. In total, 200 documents were provided to each annotator. This number was chosen based on the time taken by the annotators. The 200 documents allowed for a quick turnaround and revisions of the guidelines where required. The purpose of this was to ensure all the annotators were in agreement. At the end of each round, they provided feedback on some common false positives or ambiguous mentions, and any disagreements were discussed. Updates were made to the annotation guidelines accordingly. Interannotator agreements were calculated after each round of annotations. Once satisfactory interannotator agreement was achieved, the main annotation process commenced where each annotator was given separate sets of documents to annotate. The annotation process (Figure 1) displays the steps followed by the annotators.

**Table 3:** Examples of annotations.

Sentence	Keyword	Correct	Relevant	Anatomy	Pain character	Pain management
He likes *burning* things	Burn	No	N/A^a^	N/A	N/A	N/A
She *pain*ted a picture of the situation	Pain	No	N/A	N/A	N/A	N/A
She is in *constant pain*	Pain	Yes	Yes	N/A	Other	N/A
He suffers from *severe headaches*	Ache	Yes	Yes	Mentioned	Other	N/A
He is not on *painkillers*	Pain	Yes	Negated	N/A	N/A	Medication
Afraid I will be in *pain* if surgery is unsuccessful	Pain	Yes	No	N/A	N/A	N/A

^a^N/A: not applicable.

Annotations were marked as correct if the preannotated pain-related mention was in fact a mention of pain in the medical sense of the word. Mentions that were not related to human pain would be marked as incorrect, such as “…burn marks on the door” or “burning incense” for the pain-related term “burn” since the mention would be in relation to an inanimate object.

Correct mentions were labeled as “relevant” if they were referencing pain in a medical context, and it was the patient in question who was experiencing the pain. Some examples of mentions of pain that would be marked as “not relevant” were mentions referencing someone else’s pain, such as “his mother was always in pain,” or metaphorical or hypothetical mentions such as “fear of pain in the future” or the English phrase “sticking out like a sore thumb” for the pain-related term “sore.” Mentions were marked as “negated” if they referenced absence of pain, such as “she was not in pain,” “no pain reported,” or “he does not complain of headaches.”

Relevant mentions have 3 further potential attributes—anatomy, pain character, and pain management. If a mention of pain referenced a particular body part, such as “headache” indicating head, or “chronic back pain” indicating back, these were annotated as “anatomy mentioned.” If anatomy was not mentioned, the attribute defaulted to “N/A.”

If the pain character was referenced, it was annotated as “chronic” if the character mentioned was chronic, such as “chronic back pain” or “chronic pain,” and “other” if it was any other character of pain, such as “shooting pain…” or “throbbing ache.” If pain character was not mentioned, the attribute defaulted to “N/A.”

If pain management measures were indicated around the mention of pain, these were annotated as “medication” if there was reference to painkillers or other medications or “other” for mentions like physiotherapy, pain clinic, or massage. If pain management measures were not mentioned, the attribute defaulted to “N/A.” Some examples of annotations are listed in Table 3.

The annotations that were made during the training rounds went through a process of adjudication where a final annotation was chosen from the double-annotated mentions based on the latest iteration of the annotation guidelines. These adjudicated annotations, along with the main annotations, make the final corpus of annotated mentions. Upon completion of the annotation process, the prevalence of the different labels was examined. The final annotation guidelines have been made openly available for use by other researchers on similar projects and can be accessed in GitHub [20].

## Results

### Annotation Process

**Figure 2:**
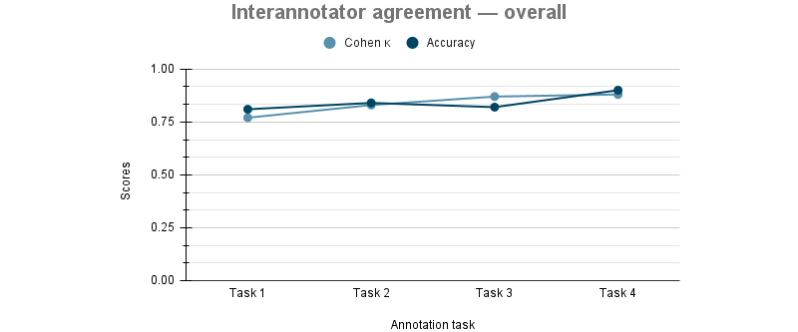
Overall accuracy and Cohen κ scores.

Four rounds of training annotation were conducted to achieve satisfactory interannotator agreement. Each annotation round consisted of about 200 documents. The corresponding number of annotations and interannotator agreements are summarized in Table 4 and Figure 2.

**Figure 3:**
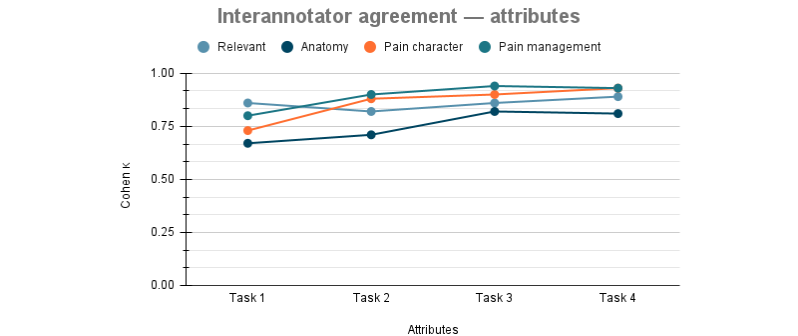
Interannotator agreement for pain attributes.

Agreements for each attribute gradually increased with each round too, as displayed in Table 5 and Figure 3. This reflects the discussions conducted with all the annotators to review any disagreements and improve the guidelines after every round of annotations.

**Table 4:** Summary of the overall annotation rounds. Agreement was calculated using the Scikit-learn accuracy and Cohen κ function [21] on the annotations.

Annotation round	Documents, n	Annotations, n	Cohen κ	Agreement (%)
Round 1	195	181	0.77	81
Round 2	195	205	0.83	84
Round 3	200	246	0.87	82
Round 4	200	297	0.88	90

**Table 5:** Summary of the interannotator agreement (Cohen κ) on attributes for annotation rounds.

Annotation round	Relevant	Anatomy	Pain character	Pain management
First	0.86	0.67	0.73	0.80
Second	0.82	0.71	0.88	0.90
Third	0.86	0.82	0.90	0.94
Fourth	0.89	0.81	0.93	0.93

**Figure 4:**
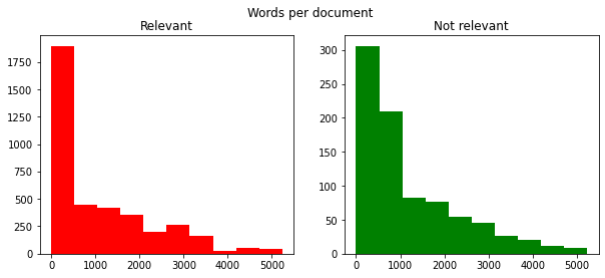
Distribution of words per document for each class.

A total of 5644 annotations were collected from 1985 documents (723 patients; summarized in Tables 6-8, and Figure 4). This includes the adjudicated annotations from the first 4 training rounds where annotators double-annotated the documents. The objective was to obtain a minimum of 975 annotations based on sample size calculations conducted following an approach proposed by Negida et al [22] but obtain more (as many as possible) if time permitted. The calculations are outlined in Multimedia Appendix 1 [22,23].

The demographic distributions of the annotation cohort were compared to that of the CRIS population, that is, all the patients within the CRIS database, and are shown in Table 9.

Most annotations (n=1857, 33%) were from patients who had a primary diagnosis of mood disorders (International Classification of Diseases—10th edition chapter F30-39; Table 10).

After a few rounds of annotations of separate documents by the annotators, another interannotator agreement check was carried out to ensure there was still good agreement among the annotators. Cohen κ score stayed at 0.88 and accuracy at 92%.

While the majority of the instances were straightforward to interpret as relevant and mentioning one or more of the attributes, some instances caused disagreements among the annotators, such as mentions of “period pain” and whether this should be considered relevant and a character of pain, since period pain has distinct characteristics, or whether it should be annotated relevant with anatomy mentioned. It was decided that such an instance would be classified as “relevant” pain with pain character labeled as “other.” Instances such as “…causing him pain” have been mentioned in situations indicating physical pain, such as “…suffering from arthritis for 20 years which is constantly causing him pain,” or referencing emotional pain, such as “…despite causing her a lot of pain, she returns to him.” It was important to consider the context of these mentions to decide whether they were physical or emotional mentions of pain. The issue of uncertainty also caused disagreement among annotators, where mentions of pain were followed or preceded by a question mark, such as “migraine?” or “?migraine.” Such instances were marked as “not relevant” since they were inconclusive. Ambiguous mentions such as “ongoing back pain” with no information on the time periods for the ongoing pain made it difficult to assess whether some mentions referred to chronic pain or not. Due to this, only instances that explicitly mentioned “chronic” were labeled as chronic pain.

**Table 6:** Annotation summary—overall.

	Values
Total number of patients whose documents were annotated	723
Total number of documents annotated	1985
Average number of words per document	1026
Average number of characters per document	6474

**Table 7:** Annotation summary—per patient.

	Values
Average number of annotations	8
Maximum number of annotations	920
Minimum number of annotations	1

**Table 8:** Annotation summary—per document.

	Values
Average number of annotations	3
Maximum number of annotations	84
Minimum number of annotations	1

**Table 9:** Patient summary compared to the CRIS^a^ population in 2009 [13].

		Annotation cohort (N=5644), n (%)	CRIS population (N=122,440), n (%)
**Age (years)^b^**			
	≤20	538 (10)	20,274 (17)
	21-40	1205 (21)	43,610 (36)
	41-60	1439 (25)	36,305 (30)
	61-80	1888 (33)	12,881 (11)
	>80	566 (10)	9370 (8)
**Gender**			
	Male	2991 (53)	60,833 (50)
	Female	2653 (47)	61,342 (50)
**Ethnicity**			
	White	3659 (65)	52,905 (68)
	Mixed	84 (1)	1026 (1)
	Black	941 (17)	16,856 (22)
	Asian	247 (4)	3299 (4)
	Other ethnic group	713 (13)	3588 (5)

^a^CRIS: Clinical Record Interactive Search.

^b^Age information was not available for 8 patients.

**Table 10:** Diagnosis summary.

ICD-10^a^ chapter	Annotations, n (%)
Mood disorders (F30-39)	1857 (33)
Anxiety and other nonpsychotic mental disorders (F40-49)	1122 (20)
Schizophrenia and other nonmood psychotic disorders (F20-29)	786 (14)
Mental disorder due to known physiological condition (F01-09)	460 (8)
Mental disorder, not otherwise specified	327 (6)
Mental and behavioral disorders due to substance use (F10-19)	311 (6)
Miscellaneous (other examination or no diagnosis)	222 (4)
Person with feared complaint in whom no diagnosis is made (Z71.1)	186 (3)
Developmental disorders (F80-89)	104 (2)
Behavioral (F50-59)	103 (2)
Behavioral and emotional disorders, childhood onset (F90-98)	85 (2)
Personality disorder (F60-69)	59 (1)
Intellectual disabilities (F70-79)	21 (<1)
Total	5644 (100)

^a^ICD-10: International Classification of Diseases—10th edition.

### Distributions of the Pain Attributes

Upon completion of all annotation rounds, the distributions of the various categories of annotations were summarized. The majority of the pain annotations were labeled as “relevant” (n=4028, 71%), followed by “not relevant” (n=859, 15%) and negated (n=757, 13%).

Among the relevant annotations, more than half had anatomy mentioned (n=2540, 63%).

Among the annotations with mentioned anatomical parts, the top 5 most common anatomical regions affected were chest (most common), followed by head, back, abdomen (including pelvis), and neck.

Similarly, among annotations where the pain character was mentioned as “chronic” or “other,” the majority (n=5000, 89%) fall under “N/A,” that is, pain character was not mentioned. Apart from “N/A,” “other” (n=487, 8%) was mentioned more frequently than “chronic” (n=157, 3%).

Among the annotations about pain character, chronic is most frequent, followed by burning, severe, uropathic, and constant.

Pain management attributes followed a similar trend where the majority were “N/A,” that is, nothing about pain management was mentioned with the annotation (n=5034, 89%). Apart from that, medication (n=422, 7%) was mentioned more frequently than “other” measures (n=188, 3%) such as referral to pain clinics and physiotherapy.

The most commonly annotated concepts within the documents were “pain” (2341 instances), “headache” (247 instances), and “painful” (206 instances).

## Discussion

The purpose of this research was to extract mentions of pain from mental health EHRs for use in research on pain and mental health. To achieve this, a lexicon of pain terms was used to identify documents that contained mentions of pain and related words. These documents were then manually annotated for whether the mentions were relevant, and if so, additional attributes were labeled. A total of 5644 annotations were collected, with over 70% (n=4028) of them belonging to the “relevant” class. Chronic was the most common pain character, and chest was the most common anatomical location annotated.

The development of this corpus has highlighted the ambiguous nature of pain, especially in mental health records, and how it could be mentioned in a variety of contextual situations. Despite this, the use of pain terms from the lexicon and achievement of good interannotator agreement have allowed for development of a corpus that is of good quality for use in further downstream tasks. Achievement of good interannotator agreement was made possible due to the methodological approach undertaken where they annotated sets of 200 documents at a time and discussed any issues and disagreements before moving on to more documents. As mentioned in the *Results* section, a variety of situations caused disagreements among the annotators. The disagreements highlighted the importance of context around the mentions of pain. Any decisions made on such examples have been stated in the annotation guidelines that were used in the development of this corpus and are important to bear in mind when developing any machine learning algorithms. The size of this corpus and the class proportions of 72/28 (relevant or not relevant + negated) are sufficiently large for use in development of various NLP applications [24,25]. Since there is some imbalance between the classes, favoring the “relevant” class, it is important to bear this in mind to ensure that any application built using this corpus performs better than a baseline of 72% accuracy. Other means of counteracting the imbalance can be used as well.

This research has been instrumental in enhancing our understanding of how pain is discussed in mental health EHRs. It has provided insights into the types of information typically associated with pain in this data source and has also shed light on the potential use of this valuable free-text information for future research. It is interesting to note that the majority of mentions of pain within these documents are relevant, and more than half of these contain information on anatomical location. Chest pain and headaches were the most frequent anatomical locations mentioned. A small portion of these relevant mentions (n=644, 16%) also contained information on the pain character and any pain management measures that might have been mentioned within the sentence. Where pain management measures were mentioned, they were mostly medications such as painkillers. Mood disorders (International Classification of Diseases—10th edition chapters F30-39) were the most common primary diagnosis within the cohort (n=1857, 33%). This could be because of the frequency of mood disorders within the CRIS database where they are the second most common primary diagnosis group [13]. As with most work that relies on clinician-recorded text, a limitation of this work is that the mentions of pain are those recorded from a clinician’s perspective and might not be truly representative of what the patient is experiencing. Also, the corpus may not be representative of the broader population since the data are from a specific population of patients from a mental health trust. It is important to consider these limitations when using these data for future work and drawing conclusions from it.

The corpus has been used for the development of an NLP application for classification of sentences as containing relevant mentions of pain (physical pain referring to the patient) or not. Four classifiers were trained (k-Nearest Neighbor [26], Support Vector Machine [27], Bidirectional Encoder Representations from Transformers—based model [28], and Self-Alignment Pretraining for BERT [29]). Self-Alignment Pretraining for BERT performed the best with an *F*_1_-score of 0.98 (95% CI 0.98-0.99). This work has been described in detail in reference [30]. The development of this corpus is promising for future work where these annotations will also be used to build an NLP application to automatically classify mentions of pain as whether they contain anatomical location or not. This will allow for extraction of data at a larger scale with this information, so further analysis and epidemiological studies can be conducted to better understand what body parts are commonly affected within different mental health diagnoses and how pain experiences might differ among different diagnosis groups. There is potential to answer many more research questions around pain and mental health, and this approach will unlock the data required to do so. There are plans to link these data with primary care records (Lambeth DataNet [31]), which will further improve the potential to answer critical research questions.

## Appendix

Multimedia Appendix 1 Sample size calculation.

## Abbreviations

CRIS: Clinical Record Interactive Search
EHR: electronic health record
NLP: natural language processing
SLaM: The South London and Maudsley

## Data Availability

The data sets generated and analyzed during this study are not publicly available. The source clinical data are accessed under the auspices of The South London and Maudsley (SLaM), the data custodian. The Maudsley CRIS (Clinical Record Interactive Search) platform provides access to anonymized data derived from SLaM’s electronic medical records within a bespoke information governance framework. These data can only be accessed by permitted individuals from within a secure firewall (ie, the data cannot be sent elsewhere). While this corpus is not publicly available, access can be requested by contacting the CRIS administrator at cris.administrator@slam.nhs.uk. The final annotation guidelines that were used to generate the annotations in this project have been made openly available for use by other researchers on similar projects, and these can be accessed on GitHub [20].
