# Supplementary material for: Development of a Corpus Annotated With Mentions of Pain in Mental Health Records: Natural Language Processing Approach
Source: JMIR Form Res. 2023 Jun 26;7:e45849. doi: 10.2196/45849 (PMC10337440; doi:10.2196/45849)
Supplement: Multimedia Appendix 1 [file formative_v7i1e45849_app1.docx]

# **Multimedia Appendix 1.** Sample size calculation.

The authors [22] propose calculating the sample size based on sensitivity and specificity of a similar algorithm from another study. A study conducted by Fernandes et al. (2018) has been used in this instance as it uses the same dataset as the current project (CRIS) and applies machine learning and rules-based methods to a text classifier [23].

Sensitivity and Specificity from the study by Fernandes et al. (2018): “Identifying Suicide Ideation and Suicidal Attempts in a Psychiatric Clinical Research Database using Natural Language Processing”:

True Positive (TP) = 381
False Positive (FP) = 7
True Negative (TN) = 33
False Negative (FN) = 79

Sensitivity (Recall) = 98.2%

Specificity = TN/TN+FP = 82.5%

Calculation:

Z = normal distribution value = 1.96 corresponds to 95% CI

W = maximum acceptable width of the 95% confidence interval = set at 10%

Sensitivity = 98.2% from above

Specificity = 82.5% from above


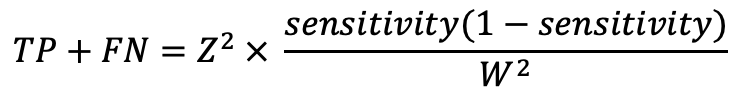


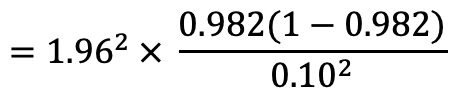


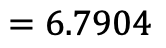


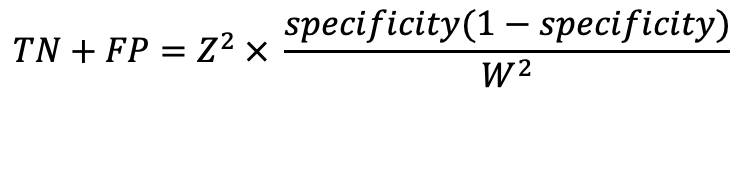


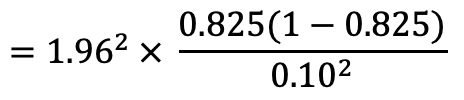


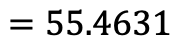


Sample size N required for sensitivity:


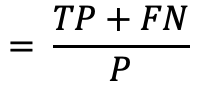


where P is the prevalence rate and stated at 5% in the same study


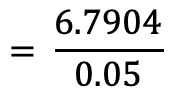


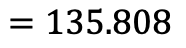


Sample size N required for specificity:


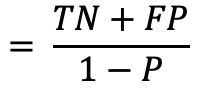


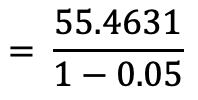


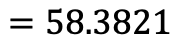


Therefore, total size = 136 + 59 = 195 mentions

This is for evaluation of a model already built, so can be assumed to be 20% of the dataset.

Therefore, main dataset sample = 195 x 5 = 975 mentions or 490 documents (with an average of ~2 mentions per document)
